# Exploring the Relationship of Well-Being and Resilience on Sense of Personal Accomplishment Among Nurse Practitioners: A Cross-Sectional Correlational Study

**DOI:** 10.1097/jnr.0000000000000707

**Published:** 2025-09-30

**Authors:** Chih-Lin Hsu, Hsuan-Man Hung, Chen-Ju Chen, Huan-Fang Lee

**Affiliations:** 1 Department of Nursing, College of Medicine, National Cheng Kung University, Tainan, Taiwan; 2 Department of Nursing, Fooyin University, Kaohsiung, Taiwan; 3 Department of Nursing, National Cheng Kung University Hospital, College of Medicine, National Cheng Kung University, Tainan, Taiwan; 4 Taiwan Holistic Care Evidence Implementation Center: A JBI Center of Excellence, Taichung City, Taiwan

**Keywords:** nurse practitioners, personal accomplishment, resilience, well-being

## Abstract

**Background:**

Nurse practitioners (NPs) play a pivotal role in controlling rising health care expenses and addressing patient preferences and emerging health disparities. However, their work may lead to work-related stress, low well-being, low resilience, and a decreased sense of personal accomplishment.

**Purpose:**

The aim of this study was to examine the relationships between well-being and resilience, respectively, with sense of personal accomplishment in the context of NPs.

**Methods:**

A cross-sectional correlational study was conducted on NPs in a medical center in southern Taiwan. The three instruments used to collect study data included the Well-being Index for well-being, the Connor–Davidson Resilience Scale (CD-RISC-25) for resilience, and the Maslach Burnout Inventory–Human Services Survey–Chinese version for personal accomplishment.

**Results:**

Good well-being, moderate resilience, and a high level of personal accomplishment were found in the 126 NP participants. Those who were married had a higher mean level of personal accomplishment, explaining 6.2% of the total variance. Well-being and two dimensions of resilience (1) personal competence and high standards and (2) positive acceptance of change and secure relationships were also identified as significant predictors of personal accomplishment, explaining 48.5% of the total variance.

**Conclusions/Implications for Practice:**

Sense of personal accomplishment is significantly impacted by well-being and several aspects (i.e., personal competence, high standards, and acceptance of change) of resilience. Health care managers should develop and implement strategies focused on reinforcing resilience and well-being to strengthen the sense of personal accomplishment in NPs.

## Introduction

Nurse practitioners (NPs), highly trained and educated health care professionals with advanced practice skills, play a vital role in the evolving healthcare landscape (Cheng et al., 2022). NPs are crucial in addressing and helping to resolve several key challenges facing the nursing profession today, including rising costs of care, expanding patient care needs and preferences, and continuing health disparities (Brom et al., 2016; Kapu et al., 2019; Tsay et al., 2023). Their responsibilities extend beyond clinical tasks, encompassing patient education and achieving a sense of professional accomplishment (Kerr & Macaskill, 2020; Henderson & Prager, 2022). Sense of personal accomplishment is defined as a feeling of incompetence and achievement at work (Maslach et al., 2001). While the NP role is recognized as essential for the delivery of high-quality and cost-effective care (Tsay et al., 2023), their expanding expectations and workloads may lead to negative consequences such as decreased motivation, low well-being, and low sense of personal accomplishment (Diehl et al., 2021; Kapu et al., 2019; Wei et al., 2021).

Well-being refers to the way in which people experience and evaluate their lives in a positive way (Tov, 2018). NPs are susceptible to work-related stress, leading to low well-being (Brom et al., 2016) and negatively impacting sense of personal accomplishment (Han et al., 2018; Wei et al., 2017), potentially increasing burnout risk (Kapu et al., 2019). When faced with overwhelming stress, inadequate coping mechanisms can lead to decreased motivation, self-doubt, and even career dissatisfaction, all of which further hinder a sense of personal accomplishment (Jose et al., 2020). High levels of well-being, encompassing positive mental health and work-life balance, have been linked to greater sense of personal accomplishment (Pastores et al., 2019), which help foster a greater perception of personal achievement, as NPs feeling mentally and emotionally well are more likely to approach their work with enthusiasm, motivation, and confidence in their ability to provide quality care (Wei et al., 2021). Conversely, low well-being can negatively impact the sense of personal accomplishment, hindering the ability to feel fulfilled in the role. Factors such as emotional exhaustion, work detachment, and reduced motivation erode NP confidence and belief in their ability to positively impact patient care, leading to decreased feelings of personal accomplishment (Guo et al., 2018).

The results of prior research indicate resilience plays a critical role in mitigating burnout and enhancing well-being among health care providers (Grabbe et al., 2021). Baugh et al. (2024) found resilience can reduce burnout among advanced practice providers, fostering a sense of personal achievement even in high-stress environments. In particular, resilience may play a significant role in moderating or mediating the impact of well-being on sense of personal accomplishment (Lee et al., 2024). Resilience, defined as the ability to adapt, bounce back, and thrive in the face of adversity (Foster et al., 2019), is another critical factor influencing perceived personal accomplishment. Resilient individuals are better equipped to cope with stressors, resist burnout, and maintain a positive outlook on their work (Gensimore et al., 2020). Therefore, resilience is essential to mitigating the negative effects of workplace challenges and enhancing well-being (Yu et al., 2019). Fostering resilience can help NPs remain motivated, focused, and determined to achieve their goals, leading to increased feelings of personal accomplishment (Guo et al., 2018). Other research results indicate individuals with a strong sense of well-being to be more likely to have the motivation, resilience, and energy needed to pursue and achieve their goals (Abraham et al., 2021; Pastores et al., 2019).

The interplay of these factors within the roles of advanced practice nursing has been explored in recent studies. For example, well-being and sense of personal accomplishment have both been linked to quality of work life and turnover intention, with resilience serving as a key buffer against negative outcomes (Lee et al., 2024). Dyrbye et al. (2020) found that advanced practice nurses with high levels of perceived personal accomplishment reported greater satisfaction with work-life integration, further emphasizing the importance of addressing these interconnected factors. Thus, the interplay among well-being, resilience, and sense of personal accomplishment is crucial for NP success. Moreover, sense of accomplishment and perceiving work tasks to be of value have both been shown to be significant predictors of intention to leave (Han et al., 2018). Although the relationship between well-being and resilience among registered nurses and physicians has been explored (Rink et al., 2022; Tzeng et al., 2023), a comprehensive understanding of these factors within the NP population is still lacking. Furthermore, although several studies suggest resilience as a mediator in the relationship between well-being and personal accomplishment (Baugh et al., 2024), this relationship has yet to be adequately examined in NPs, who face unique challenges as a subgroup of advanced practice nurses. This study was developed to address this gap by testing the hypothesis that resilience mediates the relationship between well-being and personal accomplishment. The aim of this study was to explore how well-being and resilience influence self-perceived personal accomplishment among NPs, with a particular focus on the potential mediating role of resilience.

## Methods

### Design, Sample, and Procedures

This cross-sectional correlational design study was conducted on a sample of NPs recruited from a medical center in southern Taiwan. After obtaining approval from the institutional review board (NCKUH-A-ER-109-113), the researchers distributed an anonymous questionnaire to protect the privacy of the respondents. Study purpose and procedures and the rights of participants were explained to eligible NPs, and those willing to volunteer as participants were invited to sign informed consent and obtain a copy of the questionnaire at the nursing station. The participants placed the completed questionnaire in an envelope and dropped it in a box at the station. All NPs at the targeted medical center were invited to participate. Exclusion criteria were: (1) working at the center for less than 1 month and (2) currently on leave for more than 6 months.

Using G-power software, the minimum sample size was estimated as 118 (statistical test: linear multiple regression; medium effect *f* ^2^= .15; alpha = .05; power = .80; number of predictors = 10; Polit & Beck, 2019). A total of 130 NPs were invited to participate and 126 completed the questionnaires, resulting in a response rate of 96.9%. The mean age of the participants was 38.11 years (*SD* = 6.89). Data collection occurred from December 2021 to January 2022.

### Measures

The research questionnaire consisted of two parts: demographic data and perception measurements. Demographic data included age, marital status, service unit, and self-perceived workload. The tools used in perception measurement included the Well-being Index (WBI), Connor–Davidson Resilience Scale (CD-RS), and the personal accomplishment subscale of the Maslach Burnout Inventory–Human Services Survey, Chinese version (MBI-HSS).

### Well-Being

Well-being was measured using the 9-item WBI developed by Dyrbye et al. (2010), which is designed to assess depression, anxiety, stress, fatigue, burnout, and QOL as a proxy for well-being. The first 7 items are scored either 0 or 1, and the last two items are scored between −1 and 1, giving the index a total score range of −2 to 9, with higher scores associated with a higher degree of distress, lower meaning of work, and lower satisfaction with work-life integration. The Cronbach’s alpha for the WBI in the original study was .68 (Dyrbye et al., 2010) and the index has been subsequently validated by various health care professionals worldwide (Dyrbye et al., 2016). The WBI score within the health care field may be further used to discriminate among levels of occupational satisfaction, intention to change jobs, and risk of making patient care mistakes (Dyrbye et al., 2016) and its relevance to well-being in NP has been supported by its use in studies on burnout and personal accomplishment (Dyrbye et al., 2020). The Cronbach’s alpha was .76 in this study.

### Resilience

Resilience was measured using the 25-item CD-RS, a validated tool widely used to assess resilience as a measure of stress coping capability. Developed by Connor and Davidson (2003), this scale has demonstrated robust reliability and validity across healthcare populations, including nurses and advanced practice nurses (APNs; Baugh et al., 2024; Lee et al., 2024). Each query was self-evaluated on a five-point continuum (0–4), spanning from “*none* *”* (0) to “*almost always* *”* (4). The scale is used to assess the respondent’s emotions over the preceding month, yielding a total score in the 0–100 range, with elevated scores signifying greater resilience. Notably, the scale demonstrated robust reliability during its development phase, registering coefficients of .89 (initial) and .87 (retest; Connor & Davidson, 2003). The scale assesses five subscales: toughness (8 questions), social support (7 questions), positive affect (5 questions), sense of mastery (3 questions), and spiritual support (2 questions), with scores greater than 80 indicating a good level of resilience (Mealer et al., 2017). The dimensions of resilience, particularly “personal competence” and “positive acceptance of change,” have been identified as significant predictors of personal accomplishment (Lee et al., 2024), underscoring the relevance of the CD-RS to this study. The translated Chinese version of the CD-RS was previously assessed with Cronbach alpha reliability coefficient of .95 (Wang et al., 2017) and in this study, the Cronbach’s alpha was .95 overall and .85, .89, .87, .71, and .80, respectively, for toughness, spiritual support, social support, positive affect, and sense of mastery in this study.

### Self-Perceived Personal Accomplishment

Sense of personal accomplishment was measured using the Personal Accomplishment Scale, a subscale of the MBI-HSS. The MBI-HSS, developed by Maslach et al. (2001), consists of 22 items in three dimensions. The Chinese version was verified by Lee et al. (2013). The personal accomplishment dimension includes seven items scored on a 7-point scale (0 = *never*; 6 = *every day*), with higher scores indicating a higher degree of perceived personal accomplishment. The Cronbach α value was .86 in the original Lee et al. study and .84 in this study.

### Data Analysis

IBM SPSS Statistics 22.0 (IBM Corp., Armonk, NY, USA) was used for all analysis work. Demographic variables were presented as frequency and percentage for categorical variables and as mean and standard deviation for continuous variables. Either the two-sample *t* test or analysis of variance was used to examine differences in personal accomplishment scores by categorical variables (i.e., marital status, work unit, and self-perceived workload). Pearson’s correlations were used to investigate correlations between continuous variables and personal accomplishment scores. Finally, multiple hierarchical linear regression was used to identify the predictors of perceived personal accomplishment. All results with *p* < .05 were considered statistically significant.

## Results

### Sample Characteristics

More than half of the participants were married, 56% worked in the general ward, and 98.4% reported their self-perceived workload as exceeding the medium level. The mean WBI score of 1.82 (*SD* = 2.75) indicates well-being was high among the participants. The mean resilience score of 121.98 (*SD* = 25.66) and mean medium scores for all dimensions of resilience indicate the participants had a moderate level of resilience. The mean personal accomplishment score of 35.25 (*SD* =7.49) indicates the participants perceived a high level of personal accomplishment (Table 1).

**Table 1:** Participant Descriptive Statistics (*N*=126)

Categorical Variable	*n* (%)
Marital status	
Single	55 (43.7)
Married	71 (56.3)
Work unit	
General ward	70 (55.6)
Acute care wards	38 (30.1)
Other	18 (14.3)
Self-perceived workload	
Low (1–3)	2 (1.6)
Medium (4–7)	65 (51.6)
High (8–10)	57 (45.2)
Missing data	2 (1.6)
Continuous Variable	Mean (*SD*)
Age (year)	38.11 (6.89)
Self-perceived workload	7.32 (1.38)
Sense of personal accomplishment (0–48)	35.25 (7.49)
Low well-being (-2 to 9)	1.82 (2.75)
Resilience (CD-RISC-25)	
Total score (25–175)	121.98 (25.66)
CST (8–56)	40.48 (8.82)
TTS (7–49)	32.68 (8.16)
ACC (5–35)	25.86 (5.62)
C (3–21)	13.60 (3.99)
SI (2–14)	9.60 (2.92)

*Note.* CD-RISC = Connor-Davidson Resilience Scale; CST = Personal competence, high standards, and tenacity; TTS = Trust in one’s instincts, tolerance of negative affect, and strengthening effects of stress; ACC = Positive acceptance of change and secure relationships; C = control; SI = spiritual influence.

### Correlation Among Well-Being, Resilience, and Personal Accomplishment

Unmarried participants reported significantly lower mean scores (*M* = 33.13) for personal accomplishment than their married peers (*M* = 36.89; *p* = .005). However, no significant differences were found in terms of work unit and self-perceived workload (Table 2).

**Table 2:** Scores of Personal Accomplishment on Categorical Variables (N=126)

Variable	Scores of Personal Accomplishment	
	Mean (*SD*)	*p*
Marital status		.005
Single	33.13 (7.79)	
Married	36.89 (6.86)	
Work unit		.258
General ward	34.41 (8.13)	
Acute care wards	36.89 (6.15)	
Other	35.00 (7.26)	
Self-perceived workload		.429
Low (1–3)	31.00 (0.00)	
Medium (4–7)	36.14 (7.49)	

*Note. p*<.05 indicates a significant difference in scores of sense of personal accomplishment.

As shown in Table 3, the results of the Pearson correlation test identified a relationship among age, well-being, resilience, and sense of personal accomplishment and a slightly negative correlation between sense of personal accomplishment and well-being (*r* = −.23, *p* < .05). Also, moderately positive correlations were identified between sense of personal accomplishment and, respectively, “personal competence, high standards, and tenacity” (*r* = .62, *p* < .001), “trust in one’s instincts, tolerance of negative affect, and strengthening effects of stress” (*r* = .58, *p* < .001), “positive acceptance of change and secure relationships” (*r* = .59, *p* < .001), “control” (*r* = .49, *p* < .001), and “spiritual influences” (*r* = .47 *p* < .001). However, only two dimensions of resilience, “positive acceptance of change and secure relationships” (*r* = −.20, *p* < .05) and “spiritual influence” (*r* = −.18, *p* < .05), were identified as (slightly) negatively correlated with well-being.

**Table 3:** Correlation Between Continuous Variables and Resilience Dimensions

Variable	PA	WB	R-CST	R-TTS	R-ACC	R-C	R-SI
Age	.02	.05	.06	−.08	−.03	−.07	−.03
PA		−.23^*^	.62^**^	.58^**^	.59^**^	.49^**^	.47^**^
WB			−.12	−.09	−.20^*^	−.18	−.18^*^
R-CST				.78^**^	.67^**^	.62^**^	.63^**^
R-TTS					.71^**^	.70^**^	.72^**^
R-ACC						.66^**^	.74^**^
R-C							.73^**^

*Note.* PA = Personal accomplishment; WB = Well-being; R = resilience; CST = Personal competence, high standards, and tenacity; TTS = Trust in one’s instincts, tolerance of negative affect, and strengthening effects of stress; ACC = Positive acceptance of change and secure relationships; C = Control; SI = spiritual influence.

^*^ *p*<.05. **p<.01.

Therefore, in this study, lower well-being scores (an indicator of psychological comfort) were shown to be associated with higher resilience and sense of personal accomplishment (Table 3).

### Hierarchical Linear Regression of the Relationship Among Demographic Variables, Well-Being, Resilience Dimensions, and Personal Accomplishment

Two models were established in the hierarchical linear regression analysis (Table 4). Model 1, used to examine the correlation between significant demographic factors and sense of personal accomplishment, showed marital status as a significant predictor of self-perceived personal accomplishment (β = 3.73, *p* = .008, *R* ^2^ = 6.2%). In Model 2, the two dimensions of well-being and resilience were added to Model 1. The significant predictors identified included well-being (β = −0.17, *p* = .018), resilience—personal competence, high standards, and tenacity (R-CST; β = 0.28, *p* = .018), and resilience—positive acceptance of change and secure relationships (R-ACC; β = 0.26, *p* = .025). The total explained variance was 52.0% and the adjusted variance of the explanation was 49.0% (*R* ^2^ = .52, adjusted *R* ^2^ = .49, *p* < .001).

**Table 4:** Hierarchical Linear Regression

Outcome Predictors	Personal Accomplishment			
	Model 1		Model 2	
	Beta	*p*	Beta	*p*
Constant	33.22		11.43	
Model 1				
Marital status (ref. unmarried)	3.73	.008	1.71	.118
Model 2				
Low well-being			−0.17	.02
R-CST			0.28	.02
R-TTS			0.15	.22
R-ACC			0.26	.03
R-C			0.21	.06
R-SI			−0.18	.16
*R* ^2^	.062		.517	
Adjusted *R* ^2^	.054		.485	
∆ *R* ^2^	.062		.455	
*F* change	7.40		16.65	
*p*	.008		<.001	

*Note.* CST = personal competence, high standards, and tenacity; TTS = trust in one’s instincts, tolerance of negative affect, and strengthening effects of stress; ACC = positive acceptance of change and secure relationships; C = control; SI = spiritual influence.

## Discussion

The findings of this study highlight the intricate interplay among demographic factors, well-being, resilience, and sense of personal accomplishment in NPs. The relationship between well-being and sense of personal accomplishment was shown to be significantly correlated, with specific resilience dimensions acting as significant predictors. In particular, “personal competence, high standards” and “positive acceptance of change and secure relationships” were identified as key resilience factors contributing to self-perceived personal accomplishment, which is consistent with Lee et al. (2024). Moreover, the results support the argument by Baugh et al. (2024) that resilience mediates the effects of workplace stressors and thus enhances a sense of personal accomplishment in demanding healthcare environments.

Being married was associated with a higher mean level of self-perceived personal accomplishment than being single. This finding aligns with Dyrbye et al. (2020), who suggested stable marital relationships provide emotional and social support that reduces occupational stress and fosters resilience. However, marriage may also introduce family-related stressors and increase overall workload (Akman et al., 2016), indicating the need for workplace interventions to help NPs balance professional and family demands.

The relationship between the resilience subscale “positive acceptance of change and secure relationships” and well-being among NPs was shown to be significant. This finding echoes Baugh et al. (2024), who found resilient health care providers to be better equipped to navigate workplace stressors while maintaining emotional stability, as well as a meta-synthesis by Kim and Chang (2022), which identified resilient nurses as better equipped to handle workplace stressors and maintain a positive outlook despite challenges. The underlying reasons for this relationship may include the supportive nature of secure relationships, which provide emotional and psychological backing during stressful situations. Furthermore, the ability to accept change positively fosters adaptability, allowing nurses to navigate the complexities of their roles more effectively. This adaptability not only mitigates stress but also promotes a sense of control and competence, which are crucial to maintaining mental health and job satisfaction (Huang et al., 2023). Evidence suggests that training and leadership play critical roles in developing nurse competencies with regard to accepting changes, while effective training programs can equip nurses with the skills needed to adapt to changing environments and strong leadership can provide the support and guidance necessary to navigate these changes successfully (Chen et al. 2020; Shahrbabaki et al., 2023). Given the demanding nature of NP work environments, resilience is a critical attribute essential for navigating stressors and achieving professional fulfilment (Guo et al., 2018). As team leaders, empowered NPs enhance care quality (McAllister & Brien, 2020). By leveraging positive relationships and acceptance of change, NPs can enhance teamwork and collaboration among health care providers, further contributing to a supportive work environment facilitative of overall well-being. Therefore, NPs who embrace changes in their lives and maintain stable relationships are more likely to experience a higher sense of well-being.

The evidence in this study and prior research confirms the positive role of spirituality in managing occupational stress among nurses. As noted in both Baugh et al. (2024) and Rogers et al. (2022), spiritual well-being is associated with lower burnout rates and improved job satisfaction. Spiritual development can be promoted through spiritual care programs and supportive environments, which enhance resilience and sense of personal accomplishment in nurses (Ünlügedik & Akbaş, 2023). Moreover, a sense of personal accomplishment has been closely linked to the level of spirituality development in nurses, with the level of spiritual well-being positively associated with the likelihood of experiencing satisfaction and achievement in their professional roles (Grabbe et al., 2021). These positive mindsets can provide NPs with a source of support, comfort, and guidance, thus enhancing their resilience and sense of personal accomplishment. In facing fast-changing environments and excessive workloads, well-trained NPs have a stronger capacity to control work stress and uncertainty.

Moderate positive correlations were found between sense of personal accomplishment and all dimensions of resilience, with the strongest positive correlations found for the dimensions of CST (personal competence, high standards, and tenacity) and ACC (positive acceptance of change and secure relationships). Personal competence refers to the belief in one’s abilities and skills to perform a task that requires having the necessary knowledge, skills, and abilities to effectively complete. This helps explain the reciprocal nature of the personal competence - personal accomplishment relationship. When NPs believe in their abilities and have a high level of personal competence, they are more likely to set challenging goals and engage in activities that lead to greater self-perceived personal accomplishment (Hudspeth & Klein, 2019). Similarly, the relationships between positive acceptance of change and secure relationships and, respectively, sense of personal accomplishment can also be seen as interconnected and mutually reinforced. Positive acceptance of change can help NPs approach new experiences and challenges with optimism. On the other hand, secure relationships provide a sense of trust, emotional safety, and connection with others. Positive acceptance of change may be facilitated by secure relationships, as these provide support and encouragement that contribute to feelings of personal accomplishment (Hakvoort et al., 2022).

### Limitations and Future Applications

By focusing on these relationships of well-being and resilience on sense of personal accomplishment among nurse practitioners, the study effectively addresses the research objectives and contributes valuable insights into the factors influencing sense of personal accomplishment in NPs. However, several limitations should be considered when interpreting and applying the findings. First, as a cross-sectional study, inferring causality, long-term effects, and generalizability to other populations should be done with appropriate caution. Second, sampling may have been affected by selection bias, as the inclusion criteria for this study were limited to NPs from a single medical centre in southern Taiwan. This may also impact the generalizability of the findings to other populations and settings. It is suggested that more representative populations be recruited in future studies to allow the examination of NPs across diverse settings and to further explore the correlations among well-being, resilience, sense of personal accomplishment, and emotional exhaustion. Finally, future studies may be designed to analyse the effect of mediations for sense of personal accomplishment-related variables to provide new insights.

The findings of this study offer valuable insights for future applications aimed at enhancing well-being, resilience, and a sense of personal accomplishment in NPs. Developing targeted interventions that address specific resilience dimensions such as “personal competence, high standards, and tenacity” and “positive acceptance of change and secure relationships” may be particularly effective in fostering a sense of accomplishment in NPs. Furthermore, integrating well-being promotion initiatives into existing training programs and workplace environments may create a more supportive and conducive context in which NPs can thrive. Exploring the specific role of social support and resources associated with marital status may lead to tailored interventions that leverage the strengths of strong relationships to enhance self-perceived personal accomplishment. By implementing evidence-based strategies that address these key areas, healthcare organizations can create a more supportive and fulfilling work environment for NPs, contributing to a stronger and more resilient nursing workforce.

### Conclusions

Well-being and resilience, particularly in the dimensions of personal competence, high standards, and positive acceptance of change and secure relationships, are significant predictors of self-perceived personal accomplishment. Health care managers should develop and implement effective strategies to enhance resilience and well-being, with particular focuses on personal competence, high standards, and positive acceptance of change, to strengthen personal accomplishment perceptions among NPs and reduce turnover intention.
